# Population Distribution, Settlement Patterns and Accessibility across Africa in 2010

**DOI:** 10.1371/journal.pone.0031743

**Published:** 2012-02-21

**Authors:** Catherine Linard, Marius Gilbert, Robert W. Snow, Abdisalan M. Noor, Andrew J. Tatem

**Affiliations:** 1 Spatial Ecology and Epidemiology Group, Department of Zoology, University of Oxford, Oxford, United Kingdom; 2 Biological Control and Spatial Ecology, Université Libre de Bruxelles, Brussels, Belgium; 3 Fonds National de la Recherche Scientifique, Brussels, Belgium; 4 Malaria Public Health and Epidemiology Group, Centre for Geographic Medicine, KEMRI - University of Oxford - Wellcome Trust Research Programme, Nairobi, Kenya; 5 Centre for Tropical Medicine, Nuffield Department of Clinical Medicine, University of Oxford, Oxford, United Kingdom; 6 Department of Geography, University of Florida, Gainesville, Florida, United States of America; 7 Emerging Pathogens Institute, University of Florida, Gainesville, Florida, United States of America; 8 Fogarty International Center, National Institutes of Health, Bethesda, Maryland, United States of America; University of Bristol, United Kingdom

## Abstract

The spatial distribution of populations and settlements across a country and their interconnectivity and accessibility from urban areas are important for delivering healthcare, distributing resources and economic development. However, existing spatially explicit population data across Africa are generally based on outdated, low resolution input demographic data, and provide insufficient detail to quantify rural settlement patterns and, thus, accurately measure population concentration and accessibility. Here we outline approaches to developing a new high resolution population distribution dataset for Africa and analyse rural accessibility to population centers. Contemporary population count data were combined with detailed satellite-derived settlement extents to map population distributions across Africa at a finer spatial resolution than ever before. Substantial heterogeneity in settlement patterns, population concentration and spatial accessibility to major population centres is exhibited across the continent. In Africa, 90% of the population is concentrated in less than 21% of the land surface and the average per-person travel time to settlements of more than 50,000 inhabitants is around 3.5 hours, with Central and East Africa displaying the longest average travel times. The analyses highlight large inequities in access, the isolation of many rural populations and the challenges that exist between countries and regions in providing access to services. The datasets presented are freely available as part of the AfriPop project, providing an evidence base for guiding strategic decisions.

## Introduction

Geography plays a significant role in the development process [1]. Three spatial features influence the economic development of a region: the density (e.g. agglomeration, scale economies), the distance (e.g. spatial mobility and access) and division (e.g. the spatial integration of economies). Improving access to people and markets is a key driver for development and plays an important role in poverty reduction.

Development among rural populations depends on access to markets for buying and selling goods, to water and fuel, and to various social and economic services such as education, healthcare or banking and credit [2]–[4]. The lack of a reliable transport system forces rural populations to spend a significant amount of time in travelling to meet basic needs and increases the transport costs incurred to access these services [2]–[4]. These factors often mean that isolation is seen as the main contributor to poverty according to the poor people themselves [4]. The proximity of a major settlement provides business for isolated populations, and connectivity with international and regional markets creates economic opportunities. For example, agricultural productivity is highly correlated to the proximity to urban markets in sub-Saharan Africa [5]. Accessing populations efficiently is also of key importance in public health, for delivering equitable and complete healthcare [6], for planning vaccine campaigns or distributing resources. Improving the accessibility of remote populations is an important priority for many of the Millennium Development Goal (MDG) targets, such as those focussed on eradicating extreme poverty, achieving universal primary education and developing a global partnership for development [4]. The measurement of accessibility of populations and settlements is therefore of importance in measuring progress towards achieving these goals.

Where transport infrastructures and access patterns are heterogeneous in space, population distributions and accessibility between populations should be estimated at levels of spatial detail that are similar or finer than the scales of this heterogeneity. Building development strategies and providing policy-relevant guidance therefore requires a detailed and accurate evidence base documenting the spatial distribution of populations, settlements and their interconnectivity. Existing spatial demographic datasets for the low income world, where access remains a substantial barrier to development, are generally based upon outdated and low resolution data, however [7]. Previous studies have developed accessibility indices based on household surveys [4] or more objective GIS-based approaches [8]. However, the lack of sufficiently detailed population and settlement distribution data, especially in Africa, impacts the availability and reliability of spatially-detailed accessibility information. Accurate and spatially detailed population distribution data can make accessibility measures considerably more valuable than can be obtained with aggregated population data. National census population data can be represented as continuous gridded population distribution datasets through the use of spatial interpolation algorithms. Differing projects and modelling techniques for the spatial reallocation of populations within census units have been developed in an attempt to overcome the difficulties caused by input census data of varying resolutions and provide standard global spatial datasets of population distribution. The most widely used of these are the Gridded Population of the World (GPW) [9], [10], the Global Rural Urban Mapping Project (GRUMP) [11], LandScan [12], and the United Nations Environment Programme (UNEP) Global Population Databases [13]. For a comparison of these datasets, see Tatem et al. [7]. While these represent valuable resources for a range of applications that require gridded population data, their lack of spatial detail and information on rural settlements make them unsuitable for thorough analyses of accessibility and connectivity. Moreover, the age and low spatial resolution of much of the input census data, shown to be an important factor in the accuracy of population mapping [14], also represent limitations for contemporary, national-scale analyses.

Here we develop and apply population-land cover relationship-based methods [15]–[17] to model settlement and population distributions and densities for the whole Africa at a finer spatial resolution than ever before undertaken. Based on these datasets, we analyse spatial population and settlement patterns, as well as how they are connected by existing transport networks. Given the impact that isolation has on poverty of rural populations and on the economies and health of nations, such spatial demographic datasets are a valuable resource for both research and operational applications in the development of low income countries.

## Materials and Methods

### Population distribution modelling

The methods used here to model population distribution in Africa are adapted from previous work undertaken for East Africa [15]–[17]. The methods were modified for ease of replication and to facilitate the incorporation of new data. Full details on population distribution modelling methods are presented in Text S1. Tables summarizing country-level input data are available on the AfriPop website: www.afripop.org.

Recent work showed that GlobCover was the global land cover dataset that, combined with detailed settlement extents, produced the most accurate population distribution data in an African context [17]. The GlobCover dataset was modified to accommodate the more detailed settlement extents obtained from satellite imagery and geolocated points. The GlobCover dataset was first resampled to 100 m spatial resolution, and the urban class – which typically overestimates settlement extent size [15], [17] – was removed and the surrounding classes expanded equally to fill the remaining space. The more detailed settlement extents were then overlaid onto the ‘urban class deprived’ land cover map and land covers beneath were replaced to produce a refined land cover map focussed on detailed and precise mapping of human settlements.

Human population census data, official population size estimates and corresponding administrative unit boundaries at the highest level available from the most recent available censuses were acquired for each African country. High resolution census data were available for three countries in Africa: Ghana, Swaziland and Kenya. Kenya data were also available at enumeration area level (finer than level 5) for 58 of the 69 Kenyan districts. Also obtained was a population density map of Namibia at 1 km spatial resolution (for details on the Namibia density map, see description on www.afripop.org). A table summarizing the spatial resolution, year and source of all data used is available on www.afripop.org.

The modelling method distinguishes urban and rural populations in the redistribution of populations. Major settlements have population numbers already derived and validated and this makes up 38% of the total African population. The remaining 62% rural population was redistributed using land cover-based weightings. The refined land cover data and fine resolution population data from Ghana, Kenya, Namibia and Swaziland were used to define per land cover class population densities (i.e. the average number of people per 100×100 m pixel), following approaches previously outlined [15], [17]. These land cover specific population densities were then used as weights to redistribute the rural populations within administrative units in the remaining African countries. The population sizes at the national level for each dataset were projected forward to 2010 with rural and urban growth rates estimated by the UN Population Division [18]. The GRUMP urban extents (available online at: http://sedac.ciesin.columbia.edu/gpw) were used to distinguish between urban and rural areas.

### Accuracy assessment

The accuracies of satellite-derived settlement extents used to refine the land cover dataset were assessed in different ways, as described in Text S2. Accuracy assessment of largescale population datasets is always challenging due to the use of all geographically-specific data to produce the population dataset, leaving little independent data for testing. However, simple comparison tests with existing gridded population datasets can be undertaken. Here, detailed census data from four African countries were used to assess the accuracy of our modelling method compared to the modelling method used for the construction of the most widely used population datasets: GPW [9], [10], GRUMP [11], LandScan [12] and UNEP [13].

Accuracy assessments were conducted for four countries where data on census counts or official population estimates were reported at a higher administrative unit level than used in the construction of each of the four gridded population datasets: Mali, Namibia, Swaziland and Tanzania. Population modelling was undertaken using census data at an administrative level lower than available – the same administrative level as used in the construction of GPW, GRUMP, LandScan and UNEP – and the accuracy of the resultant maps were tested with the higher level data. Comparisons were undertaken through calculation of RMSEs between the per-unit population counts in the fine resolution datasets and those estimated by the four spatial population datasets. The accuracies of the population datasets were also assessed at the pixel level using the population density map of Namibia at 1 km spatial resolution. To ensure a fair test, the dataset presented here was degraded from 100 m to 1 km resolution. Full details on the accuracy assessment tests are presented in Text S2.

### Settlement patterns, population distribution and accessibility

A variety of measures were calculated based on the population distribution dataset in order to characterize the settlement patterns, the population distribution and accessibility in Africa. First of all, we extracted the *percentage of the land surface that contains 90% of the population*. This measure emphasizes countries of highly focal population distribution and those where the population is more dispersed. Secondly, a *settlement aggregation index* was calculated based on the spatial distribution of the detailed settlement extents used as input data for the population distribution modeling. We extracted the Clark and Evans aggregation index R, which is a simple measure of clustering or ordering of a point pattern, using the spatstat package in R [19]. It is the ratio of the observed mean nearest neighbor distance in the pattern to that expected for a Poisson point process of the same intensity [20]. A value R>1 suggests ordering, while R<1 suggests clustering. A correction for edge effects was included using the cumulative distribution function method proposed by the spatstat package for non-rectangular windows. The variation of the Clark and Evans settlement aggregation index within countries was also calculated. Thirdly, we calculated the *average per-person travel time* to the nearest settlement of more than 50,000 inhabitants. Several studies showed that poverty is more strongly correlated with travel time to large settlements, rather than local markets or large capitals [21]–[24]. We combined the global map of accessibility developed by Nelson [25] with our detailed population distribution dataset to calculate the average travel time of people to the nearest settlement of more than 50,000 inhabitants. The *skewness of the travel time distribution* across the population was also calculated, in order to estimate the proportion of population residing in relatively inaccessible areas. All these measures were calculated at the national level and at the first administrative unit level.

## Results

**Figure 1:**
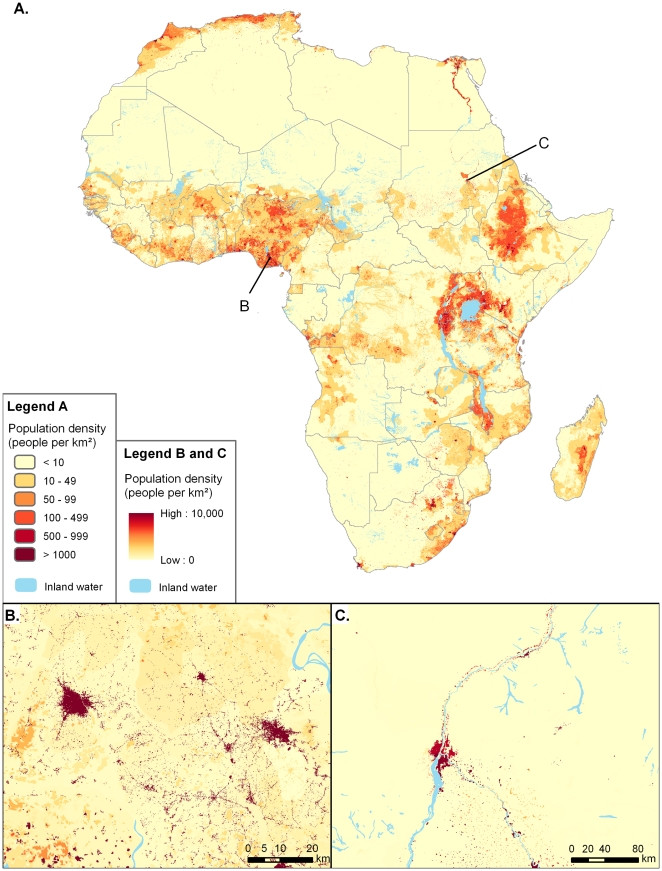
The spatial distribution of population in Africa in 2010. Maps show the original 100 m resolution dataset constructed using the methods described here. (A) Whole Africa database. (B) Close-up for a region in South-East Nigeria. (C) Close-up for the Khartoum area, Republic of the Sudan.

Gridded population datasets at ∼100×100 meters (0.000833 decimal degrees) resolution for 2010 were constructed for each mainland African country (n = 50). Two versions were produced: one with the total population adjusted to UNPD national estimates [26] and one non-adjusted (Fig. 1). Accuracy assessment results for the modelling methods used in the construction of each of the population datasets are shown in Text S2. While assessments were only undertaken for a few countries, they showed that the modelling method described here produced consistently more accurate datasets than the methods used in the construction of existing largescale gridded population data products, GRUMP, GPW, LandScan and UNEP. In some cases, the LandScan modelling method proved to be equally as accurate, but at the pixel level, LandScan was the least accurate of the population datasets tested for Namibia. The population datasets presented here aim to overcome some of the major sources of uncertainty inherent in previous mapping efforts by focussing on (i) the detailed and accurate mapping of settlements, where the vast majority of people reside, (ii) the construction of a database of more recent and detailed African administrative boundary-matched census data than has ever before been assembled (Fig. S1.1), (iii) the development of semi-automated methods that can incorporate valuable ancillary datasets on population distribution and enable ease of update.

**Figure 2:**
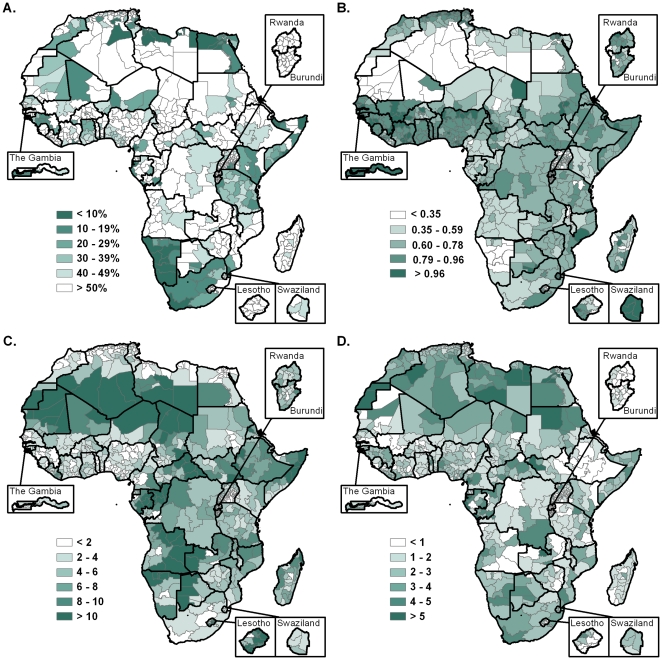
Measures for characterizing settlement patterns, population distribution and accessibility in Africa at administrative level 1. A. Percentage of land surface concentrating 90% of population. This measure emphasizes provinces of highly focal population distribution (in dark) and those where the population is more dispersed (in white). B. Clark and Evans aggregation index of settlement point patterns (<1 suggests clustering; >1 suggests ordering). C. Average per-person travel time to nearest settlement with more than 50,000 people, calculated by combining the global map of accessibility [25] with our detailed population distribution dataset. D. Skewness of the average per-person travel time across the population. A high skewness (in dark) suggests that people are concentrated in cities, whereas a low skewness (in white) suggests that a high proportion of population resides in relatively inaccessible areas.

Based on the spatially detailed population dataset described here, the spatial patterns and configurations of African populations were characterized using different spatial metrics. Fig. 2 shows different measures for characterizing settlement patterns, population distribution and accessibility in Africa. Several regions show a highly focal population distribution, with 90% of the population concentrated in less than 10% of the land surface, such as in South Africa, Namibia, and along the North and East coasts (Fig. 2a). At the regional level, Northern Africa has the more focal population distribution, with 90% of the population living in less than 8% of the territory (Table 1). Regions and countries are also highly heterogeneous in terms of settlement patterns. The Clark and Evans aggregation index is lower in Northern Africa and Namibia, showing again the highly clustered settlement and population distribution pattern in these arid regions (Table 1 and Fig. 2b). The aggregation index is however close to one in the majority of Western African administrative units, suggesting a more uniform distribution of settlements across the landscape. Southern Africa presents the highest variation between countries, with a much more aggregated settlement pattern in Namibia than in the other Southern African countries. In terms of accessibility, Fig. 2c highlights the African regions where the average travel time per person to settlements of more than 50,000 inhabitants is longest: the Sahara and Kalahari deserts, and a large part of Angola. Regional average travel times to these large settlements are however higher in Central and East Africa, with values close to 5 hours (Table 1), as a larger proportion of population resides in relatively inaccessible areas. In contrast, the average travel time is lowest in Northern Africa (lower than 2 hours) because a large proportion of people reside in very accessible areas on the Northern coast. This is also demonstrated by the skewness of the travel time distribution across the population (Fig. 2d).

**Table 1:** Settlement patterns, population distribution and accessibility measures calculated for the 5 geographical regions of Africa.

Africa region^1^	% land surface that contains 90% of the population	Aggregation index of settlements R^2^	Variance of R within region	ATT^3^	Skewness of ATT
Central	36.499	0.560	0.010	4.769	3.787
East	34.484	0.629	0.014	4.861	2.060
West	23.161	0.596	0.038	2.171	3.695
South	11.409	0.474	0.081	2.763	4.493
North	7.675	0.466	0.034	1.992	4.932

Regions defined by the United Nations (http://unstats.un.org/unsd/methods/m49/m49regin.htm).

2 Clark and Evans aggregation index calculated based on settlement point patterns. A low R value (<1) suggests that settlements are spatially clustered, whereas a high R value (>1) suggests that settlements are spatially ordered.

3 Average travel time to settlements of more than 50,000 people (hours).

**Figure 3:**
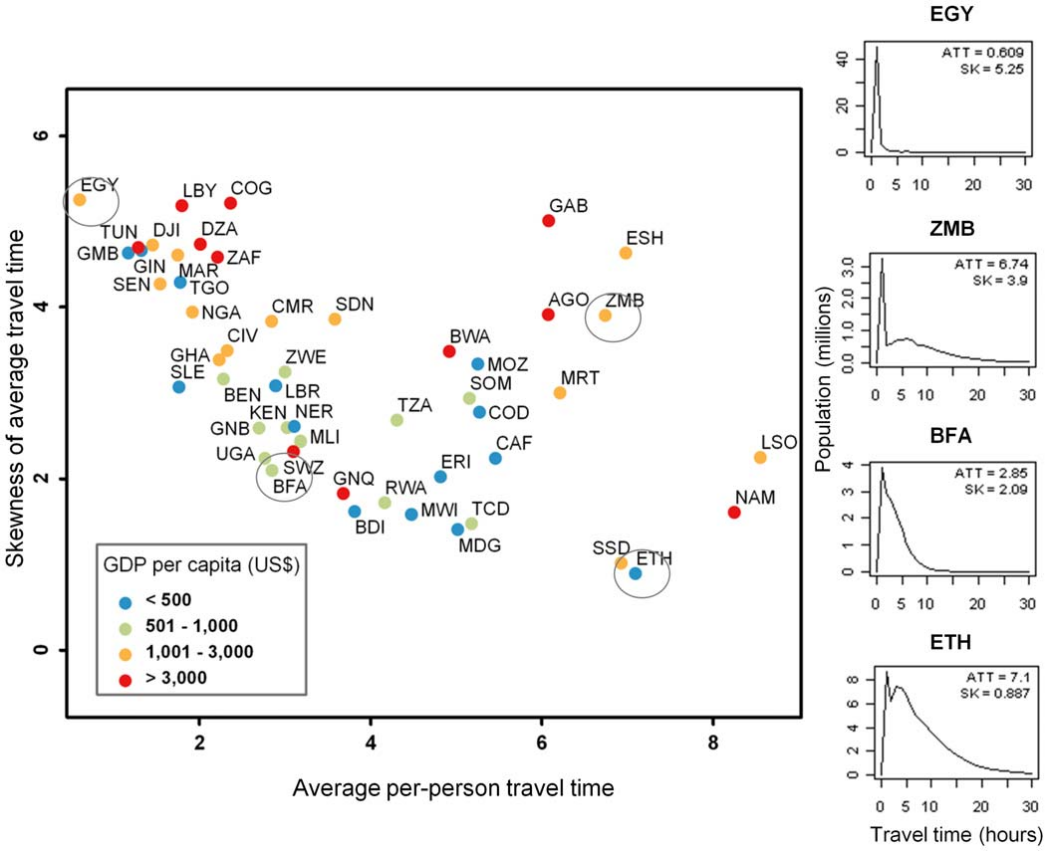
Scatterplot of the average per-person travel time versus the skewness of this average travel time. The X-axis represents the average per-person travel time to the nearest settlement with more than 50,000 people and the Y-axis is the skewness of the average travel time distribution. Examples of corresponding national travel time distribution plots are shown on the right. Colors represent the GDP per capita for the year 2010, or the year 2009 when 2010 data were not available (World Bank national accounts data, and OECD National Accounts data files: http://data.worldbank.org/indicator/NY.GDP.PCAP.CD). North and South Sudan share the same GDP per capita value, as no separate statistic is available at the moment.

A lower skewness of the travel time distribution (e.g. in Eastern Africa) shows a higher proportion of population residing in inaccessible areas. Fig. 3 shows the variation of the national average per-person travel time with the skewness of this average travel time. The scatterplot shows an exclusion zone in the bottom left corner, i.e. a country cannot combine a low average travel time with a low skewness. The relationship presents a negative trend, as a lower skewness generally means a higher proportion of people in relatively inaccessible areas. Some countries however combine relatively high travel time values with high skewness values (e.g. Gabon, Western Sahara, Angola and Zambia). These countries have a travel time plot with a very sharp peak on the left (i.e. a large part of the population is concentrated either in large settlements or areas that are very accessible to them), and smaller settlements with populations less than 50,000 generally create secondary peaks, which increase the skewness (Fig. 3). Fig. 3 also shows travel time and skewness values as a function of the GDP per capita. The GDP per capita is not significantly correlated to the average per-person travel time. However, it is generally higher in countries with higher skewness values, i.e. in countries where populations are clustered around large settlements or cities (e.g. Libya, Congo and Gabon). The poorer countries are all situated in the lower part of the graph with lower skewness values, except Gambia, Guinea and Togo. The Pearson correlation coefficient between the GDP per capita and the skewness value is not significant (Cor = 0.21; p-value = 0.137), but becomes significant if we remove Equatorial Guinea (Cor = 0.46; p-value<0.001). Equatorial Guinea is by far the African country with the highest GDP per capita (with more than 20,000 US$) mainly because of its large oil reserves and small population. Table S1 presents settlement pattern, population distribution and accessibility statistics at the national level, and Fig. S1 shows national travel time plots.

## Discussion

Rural accessibility remains a priority for poverty reduction and economic development in Africa [2]–[4]. Contemporary, detailed and regularly updated information on the spatial distribution of populations and settlements across a country and their interconnectivity and accessibility from urban areas are important for understanding a variety of social, economic, and political issues, for planning interventions and providing policy recommendations. Here we have presented the results of novel semi-automated approaches based on high resolution satellite imagery and contemporary population data to map settlements and populations across Africa, facilitating analyses of population distribution and accessibility at unprecedented levels of detail (Fig. 1).

Improving the accessibility of rural populations though transport system development is an important priority for achieving many of the Millennium Development Goal (MDG) targets, including (i) eradicating extreme poverty and hunger through improving access to inputs and markets, (ii) achieving universal primary education and gender equality through eliminating time constraints for all children to participate in education, (iii) improving child health and maternal mortality through providing an affordable access to health facilities for all households, and (iv) building a global partnership for development through reducing transport costs to access global markets [4]. Accessibility is a notion that is still rarely measured at a fine resolution, even if it is widely recognized as a fundamental indicator of economic potential. Traditionally, accessibility measures are either aggregated nationally or by administrative unit [4] or point-based using individual-level data [27]. With advances in GIS however, better accessibility measures have been developed such as friction surfaces that allows the production of largescale gridded accessibility maps [25].

The datasets outlined here provide an evidence base for quantifying isolated population sizes, monitoring changes in access and measuring progress towards the MDGs. The average per-person travel time to large settlements represents an important indicator of the accessibility to markets, while other similar indexes, such as the average travel time to schools or health facilities, can be extracted based on the datasets and approaches outlined here. Access to health facilities has been shown to be an important factor influencing maternal and infant mortality [28]–[30], while distance to schools and water sources can be important explanatory factors in poverty and food security outcomes [31]. The aggregation index of settlements can also have significant implications in terms of disease control, as a reduced connectivity between settlements and populations complicate the distribution of knowledge, preventive measures and drugs, or the organisation of vaccination campaigns. The key diagnostic measures that we provided here (Fig. 2) provide quantitative measures to guide transport infrastructure development strategies and for integrating accessibility into costing exercises. These measures are also valuable in terms of addressing equity in access to health and education, which is a central issue to the extreme poverty eradication MDG [4]. The differences exhibited between national travel time plots (Fig. 3 and Fig. S1) have significant implications in terms of equity. A more dispersed population, characterized by a low skewness of the travel time distribution, will represent a greater challenge in terms of reaching the majority of the population. For example, in many rural African regions, a large proportion of people living with HIV/AIDS have no access to treatment, simply because it is impossible to reach every infected person – this is the case in KwaZulu-Natal Province of South Africa, where populations are particularly dispersed on the territory (Fig. 2A) [32]. Accessibility indices and their variation in space and time are increasingly being used by policy makers and financiers as monitoring tools of development [8].

The proportion of the population residing in the relatively most inaccessible areas (measured by skewness of average per person travel time), shows a link to GDP (Fig. 3). Countries with a high skewness value are those where populations are clustered around big cities and therefore characterized by a high urbanization level. This confirms the hypothesis that the proportion of population living in urban areas is highly correlated with its level of income [33]. On the contrary, a country like Ethiopia, characterized by a low urbanization rate – and therefore a low skewness – and relatively high travel times, presents a low GDP per capita and faces development problems as most economic activities benefit from spatial concentration [34]. A causal link between urbanization and income level has never been proved, however. Many negative effects such as environmental degradation, pollution and population growth in slums may accompany urban growth, and urbanization may be more an indicator than an instrument of economic development [33]. Also, a high GDP per capita sometimes reflect profound inequalities between populations and economic growth does not necessarily favour the remote rural populations.

To provide reliable, contemporary and detailed measures of spatial access, transportation data must be combined with accurate and high resolution information on population and settlement distributions. This article describes the first iteration of an ongoing project entitled AfriPop (www.afripop.org), aimed at the detailed and contemporary modelling of population distribution across Africa. The modelling framework described here uses routinely collected data and semi-automated methods that can easily incorporate new data as it becomes available. Different sources of errors and uncertainties are associated with the population datasets described here, therefore, a future priority will be to incorporate uncertainty explicitly in future iterations, taking large area population distribution mapping a step further than existing approaches. Uncertainties relate to (i) the input data (ii) temporal projection and (iii) the modelling procedure used. Uncertainties associated with input data such as census data can be important, especially in low income regions where misreporting errors may be frequent [35], [36]. In addition, growth rates can vary substantially within countries, introducing uncertainties when using national estimates, and are dependent upon the urban-rural definition used. Future work will consider more sophisticated handling of these components of temporal uncertainty and its propagation through the projections. Finally, the dasymetric modelling method used here (i.e. the use of land cover data for redistributing populations) also introduces uncertainties [17]. Future iterations will focus on improvements to modelling methods, through testing alternative and promising statistical modelling methods such as boosted regression trees [37].

Access to services and resources remains a barrier to development across much of rural Africa, and the development of effective strategies to reduce this barrier requires contemporary, detailed and accurate information on population distribution and spatial accessibility upon which to base decisions. Here we have presented an Africa-wide spatial population dataset that represents an advance over other existing population distribution datasets in terms of its contemporary input data, spatial resolution and accuracy. Moreover, we have demonstrated its value in deriving spatial access metrics, and highlighted the substantial variations in settlement and population distributions and the challenges that exist between countries in improving access to services and resources. The 2010 population datasets are freely available as a product of the AfriPop Project and can be downloaded from the project website: www.afripop.org.

## Supporting Information

Figure S1 **National travel time plots.** ATT = average travel time per person to the closest settlement of more than 50,000 population. SK = skewness of average travel time. A positive skew indicates that the tail on the right side of the function is longer than the left side. Higher values indicate longer tail, i.e. a higher proportion of population located in inaccessible areas.
(TIF)

Text S1 **Population distribution modeling: material and methods.**
(DOC)

Text S2 **Accuracy assessments.**
(DOC)

Table S1 **Settlement patterns, population distribution and accessibility measures at the national level.**
(XLS)
