# Supplementary figures and images for: Population Distribution, Settlement Patterns and Accessibility across Africa in 2010
Source: PLoS One. 2012 Feb 21;7(2):e31743. doi: 10.1371/journal.pone.0031743 (PMC3283664; doi:10.1371/journal.pone.0031743)

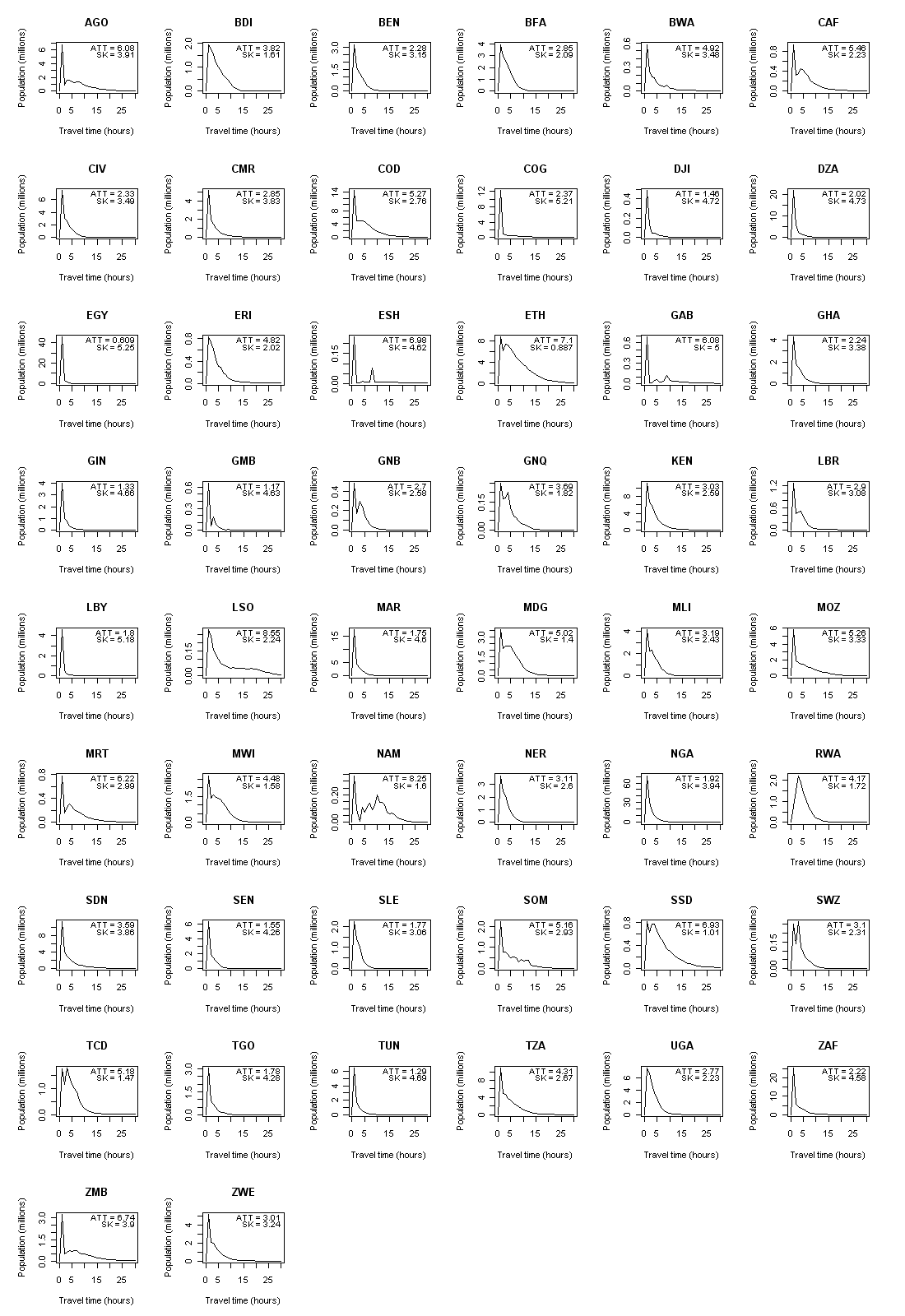

Supplement: Figure S1 — National travel time plots. ATT = average travel time per person to the closest settlement of more than 50,000 population. SK = skewness of average travel time. A positive skew indicates that the tail on the right side of the function is longer than the left side. Higher values indicate longer tail, i.e. a higher proportion of population located in inaccessible areas. (TIF) [file pone.0031743.s001.tif]
