# Supplementary material for: Population Distribution, Settlement Patterns and Accessibility across Africa in 2010
Source: PLoS One. 2012 Feb 21;7(2):e31743. doi: 10.1371/journal.pone.0031743 (PMC3283664; doi:10.1371/journal.pone.0031743)
Supplement: Text S1 — Population distribution modeling: material and methods. (DOC) [file pone.0031743.s002.doc]

## Text S1: Population distribution modeling: material and methods

**1. Land cover and settlement extents**

The GlobCover global land cover dataset is derived from a time-series of Medium Resolution Imaging Spectrometer (MERIS) images acquired from December 2004 to June 2006 (1). Recent work showed that GlobCover was the global land cover dataset that, combined with detailed settlement extents, produced the most accurate population distribution data (2). The dataset has a spatial resolution of 300 meters and is compatible with the UN Land Cover Classification System (LCCS). Linard et al. (2) showed that aggregating GlobCover land cover classes from 47 to 10 generic classes did not reduce the accuracies of output population distribution datasets. The GlobCover dataset was acquired from [http://ionia1.esrin.esa.int/], and the 10 generic GlobCover land cover classes were used as a basis for the redistribution of rural populations across Africa (described below).

The GlobCover land cover data were ‘refined’ to accommodate finer spatial resolution and more accurate information on settlement locations and extents. Two satellite-derived datasets were created for this purpose. First, a dataset depicting expert-opinion derived settlement extent outlines derived from Landsat imagery was acquired from the GeoTerraImage Consultancy [www.geoterraimage.com]. The dataset obtained covers the majority of African countries, with the exception of Burundi, Cameroon, Liberia, Rwanda and Uganda. This dataset was principally derived from 2005 Landsat imagery, through a combination of conventional expert opinion on-screen interpretation and hierarchical clustering techniques (3), often involving the use of area-specific geographic masks. Secondly, settlement maps at 30 m spatial resolution were created for the countries not covered by the dataset described above, along with Kenya and Tanzania. The methodologies used to create these datasets are based on Landsat Enhanced Thematic Mapper (ETM) and Radarsat-1 synthetic aperture radar (SAR) imagery and detailed in Tatem et al. (4). The accuracies of the satellite-derived settlement maps were assessed using approaches outlined below.

In addition to satellite-derived settlement extents described above, settlement point datasets were also used for some countries, where available, to ensure the inclusion of population clusters that were too small for identification in the satellite imagery. Geolocated point datasets that represent human presence (villages, settlements, market centres, schools, health facilities) were obtained from different sources. An approximate estimate of the precision of geolocation of these datasets was obtained through viewing each in Google Earth (5). All datasets that were insufficiently precise (i.e. datasets with many points falling well outside obvious settlements due to insufficient GPS coordinate precision) were excluded from further analysis. For those with precise geolocation, we estimated the average size of settlement extents surrounding the points. For each dataset, points that did not fall within or near (within 500m) settlements detected through the remote sensing approaches outlined above were identified. A 10% random sample of these points was created, and their average settlement sizes as depicted in Google Earth were quantified. Circular buffers of equivalent size were created around the points and the resulting layer was merged with the satellite-derived settlement extents. A table summarizing the datasets used to derive settlement extents for each country, along with data sources and buffer sizes is available on [www.afripop.org](http://www.afripop.org/). The method used for Somalia was modified due to the detailed settlement data available, and is described elsewhere (6).

The GlobCover dataset was modified to accommodate the more detailed settlement extents obtained from satellite imagery and geolocated points. The GlobCover dataset was first resampled to 100 m spatial resolution, and the urban class – which typically overestimates settlement extent size (7, 8) – was removed and the surrounding classes expanded equally to fill the remaining space. The more detailed settlement extents were then overlaid onto the ‘urban class deprived’ land cover map and land covers beneath were replaced to produce a refined land cover map focussed on detailed and precise mapping of human settlements.

**2. Population data**

Human population census data, official population size estimates and corresponding administrative unit boundaries at the highest level available from the most recent available censuses were acquired for each African country. A table summarizing the spatial resolution, year and source of all data used is available on [www.afripop.org](http://www.afripop.org/). Fig. 1 shows the spatial and temporal characteristics of the population data assembled compared with the population database used in construction of the GPW and GRUMP datasets[[1]](#footnote-2). High resolution census data were available for three countries in Africa: Ghana, Swaziland and Kenya, with average spatial resolutions (ASR)[[2]](#footnote-3) of 4.1, 2.9 and 3.6 km, respectively. Kenya data were also available at enumeration area level (finer than level 5) for 58 of the 69 Kenyan districts. Also obtained was a population density map of Namibia at 1 km spatial resolution (for details on the Namibia density map, see description on [www.afripop.org](http://www.afripop.org/)).


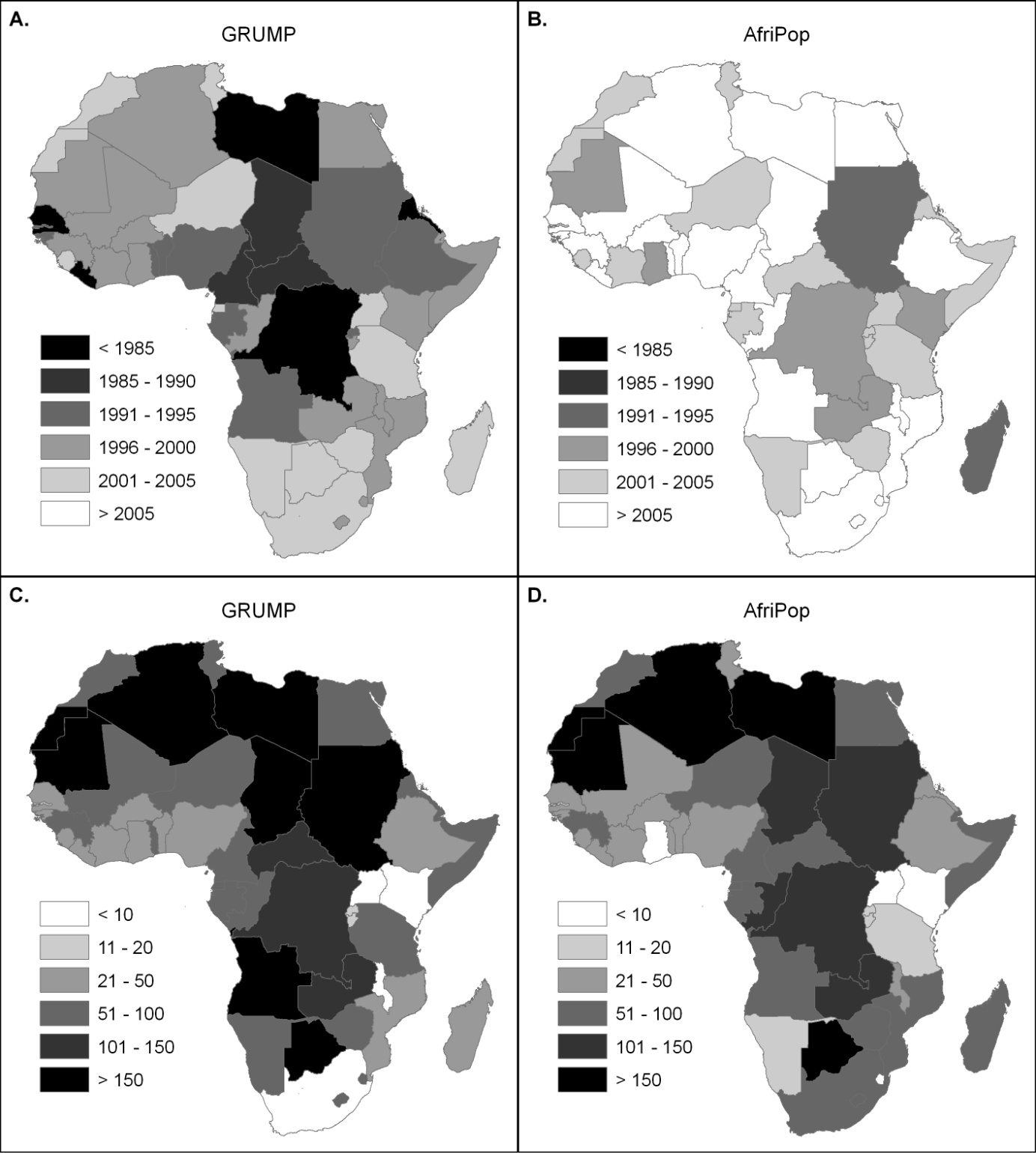


**Fig. 1:** Comparison of the spatial and temporal characteristics of population data used in the construction of the Gridded Population of the World (GPW) version 3 and the Global Rural Urban Mapping Project (GRUMP) datasets and the dataset described here (referred as ‘AfriPop’). A, B: Year of input population data. C, D: average spatial resolution (ASR) of input population data. The ASR measures the effective resolution of administrative units in kilometers. It is calculated as the square root of the land area divided by the number of administrative units (9). An ASR higher than 50 indicates an average size of administrative units upper 2500 km².

For several countries, data from the most recent census were available at a coarser administrative level than existing data from previous censuses. In such cases, we used the fine resolution data from the older census and adjusted population totals to match those estimated by the most recent census at the administrative level reported. Further, for a few countries, official government population estimates were used if the most recent census data was particularly outdated.

**3. Population distribution modelling**

The modelling method distinguishes urban and rural populations in the redistribution of populations. Major settlements have population numbers already derived and validated and this makes up 39% of the total African population. The remaining 61% rural population was redistributed using land cover-based weightings, as described below.

*City population numbers*

The proportion of people living in urban areas varies substantially from one country to the other. Given the high, and increasing, proportion of people residing in urban areas, the accurate mapping of urban extents and allocation of population to them was a priority. The GRUMP project (10) – which explicitly considered urban population in their population distribution modelling – collected information on urban extents and their population estimates from a variety of sources, primarily official statistical offices (10). This GRUMP database of geolocated populated places was used to allocate the estimated number of people to one particular city and to distribute urban and rural populations separately in administrative units were urban areas were located. Because population data came from a variety of sources, in some cases the urban area population totals exceeded those of the administrative areas in which the urban areas were located. In such cases, the most recent population estimates were used. For some countries such as Ghana and Swaziland, city population counts were not used because the resolution of census data was generally finer than city extents.

*Land cover specific population densities*

The refined land cover data and fine resolution population data from Ghana, Kenya, Namibia and Swaziland were used to define per land cover class population densities (i.e. the average number of people per 100 x 100 m pixel), following approaches previously outlined (2, 7). These land cover specific population densities were then used as weights to redistribute the rural populations within administrative units in the remaining African countries.

The extrapolation of land cover specific population densities to neighbouring regions has a limited impact on population distribution model accuracies in Kenya (2). This does not exclude accuracy losses when land cover specific population densities are extrapolated from one country to the other for large area population distribution modelling, as the relationship between population density and land cover differs from one country to the other. For example, populations are more clustered in arid environments, as was shown for Somalia (6). To account for this in the spatial extrapolation of population densities, different sets of population densities were calculated for different climate zones. The first quantitative climate classification of Köppen developed in 1900, then updated by Geiger in 1954 and 1961, is still the most frequently used (11). The Köppen-Geiger classification divides the world into five main climates: equatorial (A), arid (B), warm temperate (C), snow (D) and polar (E), with further subdivisions based on precipitation and air temperature. This map was recently digitalized and updated by Kottek et al. (11), and is available for a regular 0.5 degree latitude/longitude grid. Africa is covered by the first three climate zones: equatorial, arid and warm temperate climates (Fig. 2), and population densities by land cover class were calculated for these three zones. As shown in Fig. 2, Ghana is equatorial, Namibia is arid, Swaziland is warm temperate, whereas Kenya covers the three climate zones.

**
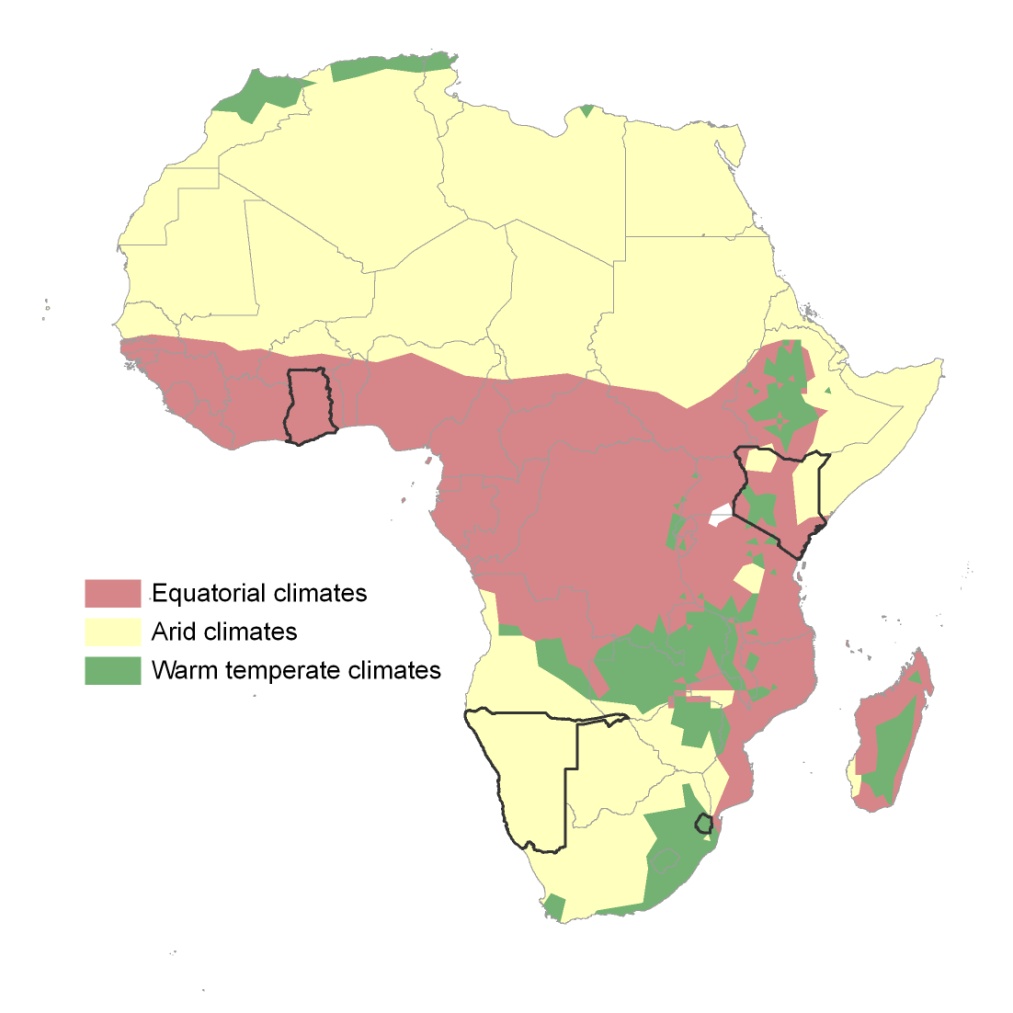
**

**Fig. 2**: Köppen-Geiger climate classification recently digitalized and updated by Kottek et al. (11). Countries highlighted (Ghana, Kenya, Namibia and Swaziland) were used to calculate land cover specific population densities (shown in Table 1) because of the very detailed census data available for these countries.

For each climate zone, Ghana, Kenya, Namibia or Swaziland census units located within the zone were first selected. The average population density of one specific land cover class was then calculated based on units that recorded this land cover class for the majority of their pixels, following methods already described in (2, 6, 7). Zeros were attributed to classes with no human habitation (water bodies and industrial areas). Per land cover class densities (shown in Table 1) were then used as weights to reallocate rural populations within administrative units in all other countries. For each administrative unit, we used weights corresponding to the climate zone that covers the majority of the unit. While such extrapolation from broad climate zone-derived weightings is not ideal, assessments showed that the approach produced more accurate representations of population distribution than existing approaches.

**Table 1**: Average population density per land cover class (from the 10 aggregated GlobCover classes) for the three main Köppen-Geiger climates present in sub-Saharan Africa (A = equatorial, B = arid, C = warm temperate). These population densities were calculated based on detailed census data from Ghana, Kenya, Namibia and Swaziland.

| **GlobCover aggregated land cover classes** | **Population density (people/km²)** | | |
| --- | --- | --- | --- |
|  | Zone A | Zone B | Zone C |
| Cultivated Terrestrial Areas and Managed Lands | 108.69 | 2.22 | 129.34 |
| Natural and Semi-natural Terrestrial Vegetation – Woody / Trees | 22.19 | 2.91 | 72.55 |
| Natural and Seminatural Terrestrial Vegetation – Shrubs | 39.67 | 1.98 | 64.97 |
| Natural and Seminatural Terrestrial Vegetation – Herbaceous | 5.85 | 2.08 | 18.22 |
| Natural and Semi-natural Sparse Terrestrial Vegetation | 5.07 | 1.75 | 36.36 |
| Natural and Seminatural Aquatic Vegetation | 78.10 | 11.25 | 53.44 |
| Bare areas | 5.30 | 1.52 | 16.02 |
| Urban settlements | | 3327.65 | | --- | | 1153.89 | 1644.40 |
| Rural settlements | 2376.54 | 332.63 | 377.10 |
| Waterbodies | 0 | 0 | 0 |
| Industrial area | 0 | 0 | 0 |

*Projection and adjustment*

The population sizes at the national level for each dataset were projected forward to 2010 with rural and urban growth rates estimated by the UN Population Division (12), using the following equation: $P_{2010}=P_{d}e^{rt}$, where *P_2010_* is the required 2010 population within a pixel, *P_d_* is the population within the same pixel at the year of the input population data, *t* is the number of years between the input data and 2010, and *r* is the urban or rural average growth rate taken from the UN World Urbanization Prospects Database, 2007 version (12). The projected output dataset totals often showed minor discrepancies with UN national population total estimates (13) because of the use of unadjusted census count data and refined urban/rural definitions. Two versions of the datasets were therefore produced: one non-adjusted and one with the total population adjusted to UNPD estimates (13).

*Two exceptions: Namibia and Angola*

High quality population density datasets were already available for Namibia and Angola and were therefore used instead of or in addition to the datasets produced using the methods outlined here. The population density dataset of Namibia at 1 km resolution was resampled to 100 m spatial resolution for consistency between countries and included unadapted in the global dataset. For Angola, detailed population density datasets at 1 km resolution were acquired for 5 main cities (Huambo, Luanda, Malanje, N’dalatando and Uíge) based on household surveys. The Angola population dataset was first constructed with the methods described above and the 5 city population datasets were then incorporated into the national dataset. A description of Namibia and Angola population datasets is available on [www.afripop.org](http://www.afripop.org/).

## References

1. Arino O et al. (2008) GLOBCOVER: The most detailed portrait of Earth. ESA Bull:25.

2. Linard C, Gilbert M, Tatem AJ (2010) Assessing the use of global land cover data for guiding large area population distribution modelling. GeoJournal 2010;76:525–538.

3. Campbell J (2007) Introduction to remote sensing, Fourth Edition. New York: Guildford Press.

4. Tatem AJ, Noor AM, Hay SI (2004) Defining approaches to settlement mapping for public health management in Kenya using medium spatial resolution satellite imagery. Remote Sensing of Environment 93:42-52.

5. Google (2010) Google Earth 6. Available: http://www.google.com/earth/index.html.

6. Linard C, Alegana VA, Noor AM, Snow RW, Tatem AJ (2010) A high resolution spatial population database of Somalia for disease risk mapping. Int J Health Geogr 9:45.

7. Tatem AJ, Noor AM, von Hagen C, Di Gregorio A, Hay SI (2007) High resolution population maps for low income nations: combining land cover and census in East Africa. PLoS One 2:e1298.

8. Tatem AJ, Noor AM, Hay SI (2005) Assessing the accuracy of satellite derived global and national urban maps in Kenya. Remote Sensing of Environment 96:87–97.

9. Balk D, Yetman G (2004) The Global Distribution of Population: Evaluating the gains in resolution refinement. Available: http://sedac.ciesin.org/gpw/docs/gpw3_documentation_final.pdf. Accessed 2011 Oct 11.

10. Balk DL et al. (2006) Determining global population distribution: methods, applications and data. Adv Parasitol 62:119–156.

11. Kottek M, Grieser J, Beck C, Rudolf B, Rubel F (2006) World Map of the Köppen-Geiger climate classification updated. Meteorol Z 15:259-263.

12. United Nations Population Division World Urbanization Prospects: The 2007 Revision Population Database. New York: United Nations. Available: http://esa.un.org/unup/. Accessed 2011 Oct 11.

13. United Nations Population Division (2008) World Population Prospects: The 2008 Revision. New York: United Nations.

1. Dates and administrative unit data obtained from: http://sedac.ciesin.columbia.edu/gpw/spreadsheets/GPW3_GRUMP_SummaryInformation_Oct05prod.xls [↑](#footnote-ref-2)
2. The ASR measures the effective resolution of administrative units in kilometres. It is calculated as the square root of the land area divided by the number of administrative units (9) [↑](#footnote-ref-3)
