# Supplementary material for: Population Distribution, Settlement Patterns and Accessibility across Africa in 2010
Source: PLoS One. 2012 Feb 21;7(2):e31743. doi: 10.1371/journal.pone.0031743 (PMC3283664; doi:10.1371/journal.pone.0031743)
Supplement: Text S2 — Accuracy assessments. (DOC) [file pone.0031743.s003.doc]

## Text S2 : Accuracy assessments

**1. Accuracy assessment of settlement extents**

**Methods**

The accuracies of satellite-derived settlement extents used to refine the land cover dataset were assessed in a variety of ways. Accuracies of settlement extent datasets produced by Tatem et al. were already assessed elsewhere using expert opinion coverage (1) and Africover settlement extents (Africover Project, Eastern Africa module, [www.africover.org](http://www.africover.org/)) (2). Landsat-derived settlement extent outlines acquired from the GeoTerraImage Consultancy [www.geoterraimage.com] were delineated using expert opinion with comparison to detailed topographic maps. These data were therefore already partially validated during the production process. Additional independent assessments with alternative data sources have been done here. Land cover data provided by the Food and Agriculture Organization of the United Nations (FAO) for 10 Eastern Africa countries (Africover Project, Eastern Africa module, [www.africover.org](http://www.africover.org/)) and Senegal (3) were obtained at full resolution (1:100,000). These data have been produced via expert interpretation of Landsat Thematic Mapper (TM) imagery and their accuracy was systematically evaluated in the field. A full reinterpretation based on all available information was undertaken to result in a rigorously defined land cover map. Landsat-derived settlement extent outlines were first compared to the FAO ‘urban area’ and ‘rural settlement’ extents for countries where FAO land cover data were available. Two sets of 10,000 points were randomly created within each country with a minimum distance of 100 m between points. The first 10,000 points were located within FAO settlement extents, and the second 10,000 points were located outside FAO settlement extents. Half of these non-settlement points were positioned randomly within 500 m buffers of FAO settlement extents to also assess the accuracy with which settlement extents were delineated by the maps. In some countries, creating 10,000 points within settlements with a minimum distance of 100 m between points was not possible and the number of settlement test points was therefore reduced. Percentage correct, Kappa and errors of commission and omission were calculated (4).

A further approach to accuracy assessment was undertaken through randomly selecting 300 settlement extents from the entire settlement extents database and overlaying these onto Google Earth (5), where the highest spatial resolution images were available. This enabled calculation of the user’s accuracy (and commission error), i.e. the probability that a settlement extent actually represents a settlement on the ground. Selected settlements positioned in areas where the distinction between settlements and non-settlements was not certain because high resolution images were not available were removed from the database (94 out of 300 settlements).

Finally, the critical size of settlements required for them to be included in the Landsat-derived settlements database was evaluated using SPOT5 data for a study area of 1930 km² in North Benin (Alibori department). Villages were visually delineated based on a SPOT5 image at 5 meters resolution acquired on 10 January 2010. The purpose was to identify and delineate every settlement for very high resolution population distribution modelling, which make these very detailed data ideal for testing our Landsat-derived settlement extents. Settlement extents derived from SPOT were compared to settlement extents derived from Landsat and a critical size under which settlements were not detected using the Landsat data was identified.

**Results**

Contemporary population data and land cover data refined with Landsat-derived settlement extents were combined to estimate population distribution across Africa at an unprecedented level of detail. The Landsat-derived settlement data created and used in dataset construction showed an overall accuracy (Kappa) of 0.63, ranging from 0.45 for the United Republic of Tanzania to 0.91 for the Democratic Republic of the Congo, when using expert-derived FAO ‘urban’ and ‘rural settlement’ extents as a reference (Table 1). Non-settlement test points were mapped more accurately than the FAO-defined settlement points, with overall percentages correct of 92.37% and 69.40%, respectively. All accuracies therefore indicated a good to excellent agreement. As shown in Table 1 and illustrated in Fig. 1, the Landsat-derived settlement extent dataset used in the population map production (Fig. 1A) contains almost 18 times more individual settlement extents than the FAO dataset (Fig. 1B), and 84 times more than the urban extents dataset used in the construction of GRUMP (Fig. 1C). Analyses showed a high likelihood that the Landsat-derived settlement extents represented settlements on the ground, with a user’s accuracy of 0.89 and a commission error of 0.11. In a small study area in North Benin, 144 independent settlements were identified visually using detailed SPOT5 images at 5 meters resolution. Only 15 Landsat-derived settlement extents covered the same area. SPOT-derived settlements smaller than 0.3 km² were never detected using Landsat images. However, 15 out of the 17 settlements bigger than 0.3 km² (88%) were also detected using Landsat images, suggesting 0.3 km² in this region at least, as a critical size for settlements to be included in the Landsat-derived settlement database.

**Table 1** : Accuracy statistics for the Lansat-derived settlement extent data used in the land cover map refinement using land cover data provided by the Food and Agriculture Organization of the United Nations (FAO) for 10 Eastern Africa countries (Africover Project, Eastern Africa module, [www.africover.org](http://www.africover.org/)) and Senegal (39). C = Error of commission, O= Error of omission.

|  | No. FAO settlement polygons | No. Landsat-derived settlement polygons | No. test points settlement/non-settlement | Settlement percentage correct | Non-settlement percentage correct | Kappa | Settlement C (%) | Settlement O (%) | Non-settlement C (%) | Non-settlement O (%) |
| --- | --- | --- | --- | --- | --- | --- | --- | --- | --- | --- |
| Dem. Rep. Congo | 445 | 48,106 | 10,000/10,000 | 93.31 | 97.42 | 0.9073 | 0.0269 | 0.0669 | 0.0643 | 0.0258 |
| Egypt | 1,080 | 9,698 | 10,000/10,000 | 82.22 | 88.35 | 0.7057 | 0.1241 | 0.1778 | 0.1675 | 0.1165 |
| Eritrea | 100 | 526 | 2,291/10,000 | 65.26 | 93.76 | 0.6077 | 0.2945 | 0.3474 | 0.0783 | 0.0624 |
| Kenya | 350 | 1,659 | 7,587/10,000 | 55.34 | 92.52 | 0.4996 | 0.1512 | 0.4466 | 0.2680 | 0.0748 |
| Senegal | 2,302 | 5,375 | 10,000/10,000 | 62.90 | 97.37 | 0.6027 | 0.0401 | 0.3710 | 0.2759 | 0.0263 |
| Somalia | 15 | 516 | 2,034/6,652 | 83.33 | 87.79 | 0.6581 | 0.3239 | 0.1667 | 0.0549 | 0.1221 |
| Sudan | 598 | 20,813 | 10,000/10,000 | 60.84 | 91.44 | 0.5228 | 0.1233 | 0.3916 | 0.2998 | 0.0856 |
| Un. Rep. Tanzania | 607 | 9,729 | 10,000/10,000 | 56.51 | 88.74 | 0.4525 | 0.1662 | 0.4349 | 0.3289 | 0.1126 |
| Total | 5,497 | 96,422 | 61,913/76,651 | 69.40 | 92.37 | 0.6305 | 0.1199 | 0.3060 | 0.2111 | 0.0763 |


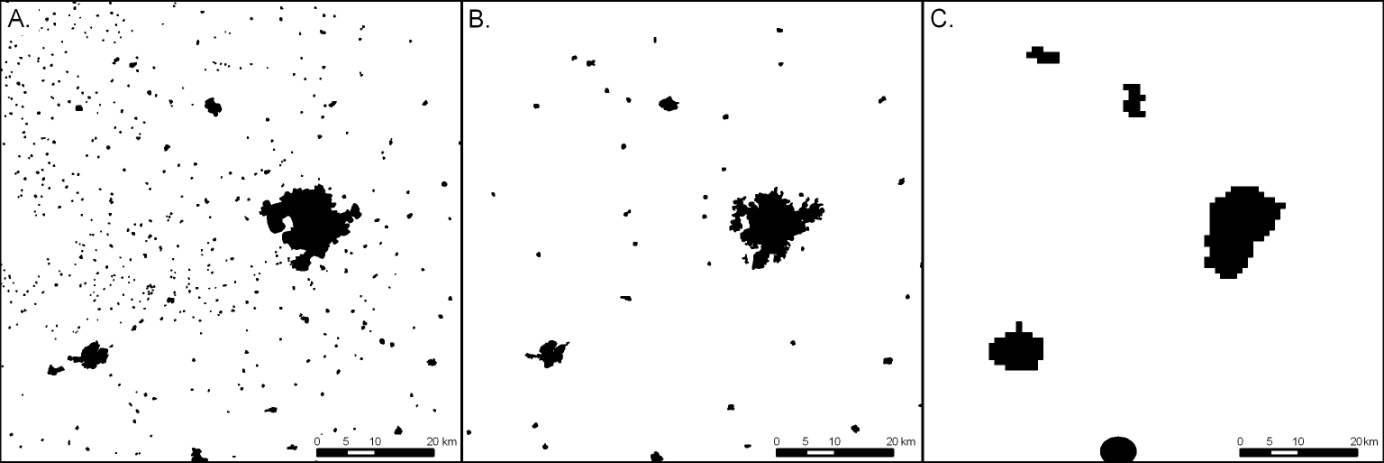


**Fig. 1**: Settlement extents in the region of Touba, Senegal, according to (A) the LandSat-derived settlement extents used in the construction of the population dataset outlined here, (B) the Food and Agriculture Organization of the United Nations (FAO) land cover dataset (39), and (C) the Global Rural Urban Mapping Project (GRUMP) urban extents data (available from <http://sedac.ciesin.columbia.edu/gpw/index.jsp>). Settlements are shown in black, whereas non-settlement areas are in white.

**2. Accuracy assessment of modelling methods**

**Methods**

Accuracy assessment of largescale population datasets is always challenging due to the use of all geographically-specific data to produce the population dataset, leaving little independent data for testing. However, simple comparison tests with existing gridded population datasets can be undertaken. Here the accuracy of our modelling method was compared to the modelling method used for the construction of the most contemporary and widely used population datasets: GPW (6, 7), GRUMP (8), LandScan (9) and UNEP (10).

Accuracy assessments were conducted for four countries where data on census counts or official population estimates were reported at a higher administrative unit level than used in the construction of each of the four gridded population datasets: Mali, Namibia, Swaziland and Tanzania. For these four countries, the construction of existing population datasets was based on population data at the same administrative level (Table 2A). The same population data were used to construct population distribution datasets based on the methods described above for these four countries in order to isolate input-dependent uncertainties from the analysis. Population modelling was undertaken using census data at an administrative level lower than available, and the accuracy of the resultant maps were tested with the higher level data (Table 2B). All the datasets were projected forward to a common year (the year of detailed population data) using UN rural and urban growth rates (11). Table 2 shows that population data used in construction of the existing population datasets and the data for assessment for the four countries cover a wide range of spatial resolutions and population per administrative units. Comparisons were undertaken through calculation of RMSEs between the per-unit population counts in the fine resolution datasets and those estimated by the four spatial population datasets. Statistical analyses including analyses of variance and Tukey’s honest significant difference tests were also performed on the differences between observed and predicted values for each administrative unit (in absolute values) to test for differences between the methods used (the one described here and the methods used in the construction of GRUMP, GPW, LandScan and UNEP) and between countries. The Tukey’s honest significant difference statistical test was used to identify which means were significantly different from the others.

**Table 2 :** (A) Characteristics of input population data used in the construction of the Global Rural Urban Mapping Project (GRUMP), the Gridded Population of the World (GPW), the United Nations Environment Programme Global Population Databases (UNEP) and LandScan for the four countries analysed. The same data were used to construct an AfriPop dataset for the accuracy assessment of modelling methods. (B) Characteristics of population data used for the accuracy assessment.

**A.**

|  | Administrative level name | No. of units | Year | ASR (km) | Average population per unit (thousands) |
| --- | --- | --- | --- | --- | --- |
| Mali | Cercle | 272 | 1998 | 68.02 | 36.07 |
| Namibia | Constituency | 102 | 2001 | 90.09 | 18.20 |
| Swaziland | Region | 4 | 1997 | 65.85 | 241.46 |
| Tanzania | District | 90 | 2002 | 102.59 | 381.93 |

B.

|  | Administrative level name | No. of units | Year | ASR (km) | Average population per unit (thousands) |
| --- | --- | --- | --- | --- | --- |
| Mali | Commune | 687 | 2009 | 42.80 | 21.12 |
| Namibia | Enumeration Area | 4072 | 2001 | 14.26 | 0.46 |
| Swaziland | Enumeration Area | 2076 | 2007 | 2.89 | 0.49 |
| Tanzania | Ward | 2806 | 2002 | 18.37 | 12.25 |

Finally, the accuracies of the population datasets were also assessed at the pixel level using the population density map of Namibia at 1 km spatial resolution. For this purpose, population data at the enumeration area level (ADM-4) were used to construct a population distribution dataset for Namibia based on the methods described above. We measured grid-based differences – as described in (12) – between the reference map and population datasets (GRUMP, LandScan, GPW and UNEP, and the dataset constructed using methods described here). Pearson correlation coefficients and RMSEs were extracted from per-pixel absolute differences between observed and predicted values.

**Results**

Fig. 2 shows a visual comparison between the dataset outlined here, GRUMP and LandScan. In the GRUMP dataset, the construction methodology means that population is concentrated into a few major urban areas and areal weighted in the remaining of the districts (21), whereas the dataset outlined here shows scattered population clusters in rural areas. In the LandScan dataset, the construction methodology means that populations are clustered around roads and less concentrated in villages and towns, but more diffuse in rural areas.

Accuracy assessments of the modelling methods used in the construction of the population datasets are shown in Fig. 3 and Table 2. For three (Namibia, Swaziland and Tanzania) out of four countries where detailed census data were available, the modelling method outlined here produced the most accurate dataset. For Mali, LandScan and the dataset constructed using methods described here were more accurate than the other population datasets tested, with root mean square errors (RMSEs) of 13,318 and 13,454 people, respectively. The relative difference in resolution between the two administrative levels (the one used for modelling and the one used for testing) was lowest for Mali (Table 1). An analysis of variance incorporating the population distribution modelling method used and the country as factors confirmed that the choice of population distribution modelling method had a significant impact on the RMSE (*F* value = 9.41; p < 0.0001). The significant interaction between the two factors showed that the effect of the method differed according to the country (*F* value = 9.91; p < 0.0001). The Tukey’s test confirmed significant differences between the predictions of the dataset constructed using methods described here and other modelling methods, with the exception of LandScan. Finally, Table 3 shows that at the pixel level, values of the dataset described here are more correlated to the population density map of Namibia at 1 km spatial resolution used here as a reference. GRUMP showed a lower RMSE value, but was closely followed by the dataset described here, GPW and UNEP, whereas LandScan showed a substantially higher RMSE.


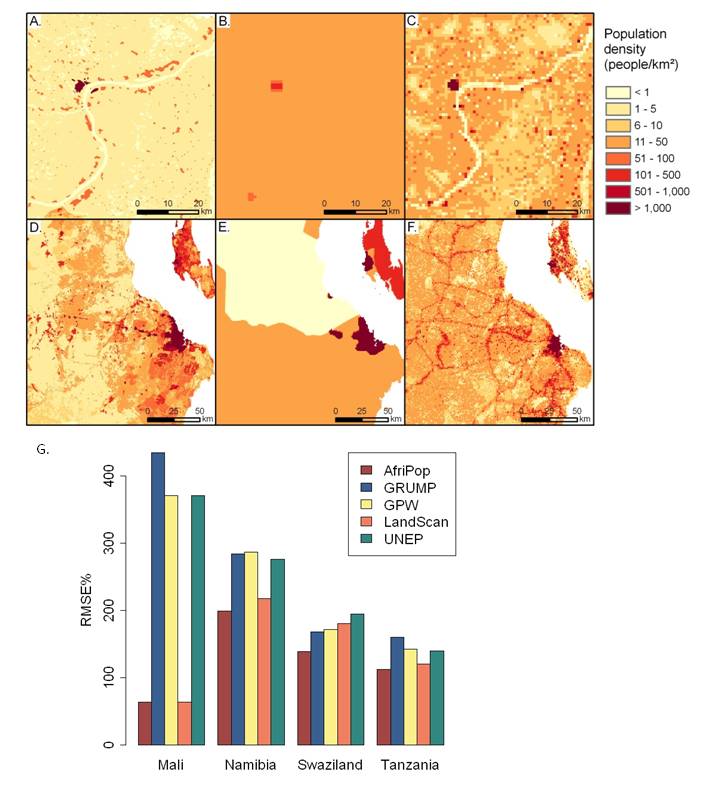


**Fig. 2**: Comparison of the dataset described here with GRUMP and LandScan gridded population datasets. Upper figures show the North-East region of Guinea, along the Niger river and lower figures show the region of Dar es Salaam, United Republic of Tanzania using (i) the dataset described in this paper (A and D), (ii) GRUMP (B and E) and LandScan (C and F). The dataset described in this paper (A and D) shows population counts per 100 x 100 metres grid squares, whereas GRUMP (B and E) and LandScan (C and F) show population counts per 1 km x 1 km grid squares.

**Fig. 3**: Results from the accuracy assessment of modelling methods performed for four countries (Mali, Namibia, Swaziland and Tanzania) where data on census counts or official population estimates reported at a higher administrative unit level than used in the construction of each of the existing gridded population datasets. Population modelling was undertaken using census data at an administrative level lower than available, and the accuracy of the resultant maps were tested with the higher level data. Root mean square errors (RMSE) are expressed as a percentage of the mean population size of the administrative level (RMSE%). The dataset described in this paper is referred as ‘AfriPop’.

**Table 2** : Results from the accuracy assessment of modelling methods performed for four countries (Mali, Namibia, Swaziland and Tanzania) where data on census counts or official population estimates reported at a higher administrative unit level than used in the construction of each of the existing gridded population datasets. Population modelling was undertaken using census data at an administrative level lower than available, and the accuracy of the resultant maps were tested with the higher level data. Root mean square errors (RMSE) are also expressed as a percentage of the mean population size of the administrative level (RMSE%). The dataset described in the paper is referred as ‘AfriPop’.

|  |  | **AfriPop** | **GRUMP** | **GPW** | **LandScan** | **UNEP** |
| --- | --- | --- | --- | --- | --- | --- |
| Mali | RMSE | 13,453.84 | 91,707.95 | 78,216.48 | 13,318.13 | 78,281.57 |
|  | RMSE% | 63.71 | 434.25 | 370.37 | 63.06 | 370.68 |
| Namibia | RMSE | 890.91 | 1,274.66 | 1,284.76 | 975.82 | 1,238.16 |
|  | RMSE% | 198.58 | 284.11 | 286.36 | 217.50 | 275.98 |
| Swaziland | RMSE | 680.41 | 822.10 | 842.48 | 884.80 | 953.48 |
|  | RMSE% | 138.69 | 167.58 | 171.73 | 180.36 | 194.36 |
| Tanzania | RMSE | 13,723.39 | 19,606.98 | 17,456.35 | 14,665.97 | 17,078.97 |
|  | RMSE% | 112.03 | 160.06 | 142.50 | 119.72 | 139.42 |

**Table 3**: Results from the accuracy assessment of existing population datasets using the Namibia density map as a reference. The absolute value of the difference between the reference map and population datasets were calculated for every 1 km x 1 km grid square. Pearson correlation coefficients (COR) and root mean square errors (RMSE) were extracted from these pixel-level difference values. The dataset described in this paper is referred as ‘AfriPop’.

|  | **AfriPop** | **GRUMP** | **GPW** | **LandScan** | **UNEP** |
| --- | --- | --- | --- | --- | --- |
| COR | 0.53 | 0.35 | 0.30 | 0.35 | 0.30 |
| RMSE | 38.32 | 38.19 | 39.60 | 50.24 | 39.62 |

**Discussion**

The validation of largescale population maps is often problematic as no independent datasets exist that can serve this purpose over wide areas. Here we assessed the accuracy of modelling methods used in the construction of various population datasets, including the one described here, and therefore compared process-dependent uncertainties. Ideally, a definitive answer to the question of which modelling approach produces superior population distribution mapping accuracy would provide valuable guidance on choosing datasets. While assessments here were only undertaken for a few countries, they showed that the modelling method described here produced consistently more accurate datasets than the methods used in the construction of existing largescale gridded population data products, GRUMP, GPW, LandScan and UNEP. In some cases, the LandScan modelling method proved to be equally as accurate, but at the pixel level, LandScan was the least accurate of the population datasets tested for Namibia. Moreover, the dataset described here is based on a very detailed mapping of settlement extents. Results showed that the settlement extents used were more accurate and detailed than any other database available at the continental level. Such spatially detailed data enable analyses to be undertaken at new refined spatial scales. In addition, census data used in the construction of the population dataset outlined here were in many cases around 10 years newer than the census data used for the construction of other existing population datasets. We have aimed to use clear and relatively simple methodologies, focusing efforts on factors known to strongly determine modelling accuracies: obtaining high resolution, contemporary census data, and the detailed mapping of settlements. Deciding among the datasets remains challenging, but transparent methodologies and clear documentation of input data make those datasets more suited to enabling researchers to understand and quantify the uncertainties inherent in them (13).

**References**

1. Tatem AJ, Noor AM, Hay SI (2005) Assessing the accuracy of satellite derived global and national urban maps in Kenya. Remote Sensing of Environment 96:87–97.

2. Tatem AJ, Noor AM, von Hagen C, Di Gregorio A, Hay SI (2007) High resolution population maps for low income nations: combining land cover and census in East Africa. PLoS One 2:e1298.

3. Leonardi U (2008) Senegal Land Cover Mapping. Food and Agriculture Organization of the United Nations. Available: http://www.glcn.org/downs/prj/senegal/Sen_lc_report_dec08.pdf. Accessed 2011 Oct 11.

4. Congalton RG (1991) A review of assessing the accuracy of classifications of remotely sensed data. Remote Sensing of Environment 37:35-46.

5. Google (2010) Google Earth 6. Available: http://www.google.com/earth/index.html.

6. Balk D, Yetman G (2004) The Global Distribution of Population: Evaluating the gains in resolution refinement. Available: http://sedac.ciesin.org/gpw/docs/gpw3_documentation_final.pdf. Accessed 2011 Oct 11.

7. Deichmann U, Balk D, Yetman G (2001) Transforming population data for interdisciplinary usages: From census to grid. Washington DC: Center for International Earth Science Information Network.

8. Balk DL et al. (2006) Determining global population distribution: methods, applications and data. Adv Parasitol 62:119–156.

9. Dobson JE, Bright EA, Coleman PR, Durfee RC, Worley BA (2000) LandScan: a global population database for estimating populations at risk. Photogramm Eng Remote Sensing 66:849–857.

10. Deichmann U (1996) A review of spatial population database design and modeling. Santa Barbara, California, USA: National Center for Geographic Information and Analysis (NCGIA), University of California, Santa Barbara (UCSB).

11. United Nations Population Division World Urbanization Prospects: The 2007 Revision Population Database (United Nations, New York) Available: http://esa.un.org/unup/. Accessed 2011 Oct 11.

12. Sabesan A et al. (2007) Metrics for the comparative analysis of geospatial datasets with applications to high-resolution grid-based population data. GeoJournal 69:81–91.

13. Tatem AJ, Campiz N, Gething PW, Snow RW, Linard C (2011) The effects of spatial population dataset choice on population at risk of disease estimates. Popul Health Metr 9.
